# Comfort during side posture lumbopelvic manipulation in a low back pain population—effects of a typical versus modified flexed lumbopelvic position: a crossover randomized control trial

**DOI:** 10.1186/s12998-025-00621-z

**Published:** 2025-11-28

**Authors:** Simon Wang, Erinn McCreath Frangakis, Martha Funabashi, Sheilah Hogg-Johnson

**Affiliations:** 1 Canadian Memorial Chiropractic College, 6100 Leslie St., Toronto, ON M2H 3J1 Canada; 2 Université du Québec À Trois‐Rivières, Trois‐Rivières, QC Canada; 3 Dalla Lana School of Public Health, University of Toronto, Toronto, Canada; 4 Institute for Disability and Rehabilitation Research, Ontario Tech University, Oshawa, ON Canada

**Keywords:** Spinal manipulation, Discomfort, Low back pain, Subgroup, Patient comfort

## Abstract

**Background:**

Literature on low back pain (LBP) treatment suggests not all LBP is the same and patients with extension- or flexion-related LBP may benefit from different strategies. This study’s objective was to evaluate the effect of participant positioning when delivering spinal manipulation on reported immediate comfort, in individuals with LBP.

**Methods:**

This a randomized crossover trial. Volunteer adult participants with LBP were recruited from a chiropractic college campus clinic to receive two procedures in random order: 1. Standard side posture lumbopelvic manipulation (SPLM) and 2. Modified flexed lumbopelvic manipulation. The modified position was similar to the standard, but with increased hip and lumbopelvic flexion. Participants were not blinded. The primary outcome of self-reported comfort was recorded using a 0–10 scale. Paired t-tests were used to compare mean comfort scores, and a minimal clinically important difference of 2.0 was used. Secondary analysis examined correlations between comfort during active range of motion (AROM) and the two SPLM procedures. An exploratory analysis comparing within-participant differences was examined. All reported harms were mild in nature (e.g. discomfort).

**Results:**

Forty participants were recruited (mean 25.5 years of age, 75% female). 20 participants started with the Standard SPLM and 20 started with the Modified Flexed SPLM. No differences in comfort were found between the Standard SPLM (N = 40) versus Modified flexed SPLM (N = 40), (mean (SD) = − 0.01 (2.3), Effect Size Cohen’s *d* = − 0.004 95% CI (− 0.32, 0.32)). No correlations were found between the comfort during AROM and the two SPLM procedures. Observing within-participant differences for individual comfort, 14 participants had a clinically significant difference ≥ 2. Specifically, 6 participants had lower scores with Standard SPLM and 8 participants had lower scores with Modified flexed SPLM.

**Conclusion:**

Comparing comfort during a Modified flexed SPLM to the Standard one revealed no difference for a population with LBP. However, some individuals did demonstrate a comfort preference for a Standard versus a Modified flexed position. Future studies should examine other variations of manipulation and particular subgroups of individuals with LBP (e.g. direction related) that experience discomfort during manipulation.

## Background

Low Back Pain (LBP) is a major healthcare challenge worldwide. It is a highly prevalent condition affecting a large part of the population, with a lifetime prevalence estimated at 50–84% [7, 29, 36]. It also results in a very high societal burden. According to the Global Burden of Disease Study 2021, LBP is one of the leading causes of non-fatal health loss, affected 619 million people globally in 2020, and is the number one cause of disability in the world [16].

Several conservative and nonpharmacological interventions are commonly used to manage LBP including education, exercise, and manual therapy. Additionally, previous studies investigating effective treatment for LBP have shown that not all LBP is homogenous and that different patients will respond better to different interventions [6, 8]. Specifically, patients subgrouped by extension-related or flexion-related LBP have been described to potentially benefit from tailored treatment suggestions to help avoid exacerbating their symptoms [14, 33, 34].

Spinal manipulation therapy (SMT) is a manual therapy technique commonly used to manage LBP and recommended by several clinical practice guidelines [5, 10, 11]. Clinical prediction rules (CPR) have been suggested for patients who, based on their clinical presentation, may respond better to lumbar SMT than others [18, 19]. However, studies attempting to validate low back pain CPRs are limited and the current body of evidence makes it difficult to differentiate between predictors of response to treatment and general predictors of positive outcome [23, 35] A predictive model using demographics, patient history, expectations, reaction to lumbar extension and the STarT back tool, suggests that patients who benefit from SMT are different from those who do not [22].

Health professions that treat people with back pain are known to utilize SMT, with chiropractors delivering the majority of those techniques [13, 32]. Comfort during SMT has been identified as an important prerequisite to performing SMT [21, 26] and has been associated with a positive response to treatment [27]. From the patient perspective, Wensley and colleagues [37] reported that comfort is a defining aspect of the patient experience. It has also been reported that chiropractic students experience pain and injury while learning SMT, with the most common site being the lumbopelvic region [25]. Bell and colleagues [4] suggested that hip position can affect the amounts of extension and rotation of the lumbar region. However, the effects of modifying lumbopelvic positioning during side posture lumbopelvic manipulation (SPLM) on people with low back pain has yet to be reported.

A recent study by the primary investigator compared discomfort scores during SMT setup for a traditional side posture hypothenar/ilium push manipulation and a modified version of the same manipulation with a flexed lumbopelvic region. They found a 53% prevalence of discomfort during the traditional setup, and a 64% prevalence of discomfort during the modified flexed setup, in a healthy young adult population. Additionally, some subjects had a preference (less discomfort) for the standard setup while others had less discomfort during the modified flex setup [28]. However, it remains unknown if similar findings would be observed in a symptomatic population.

This study examined the effects of two SPLM procedures on reported comfort, in a symptomatic low back pain adult population. A secondary question on the relationship between directional comfort and the two SPLM procedures was also examined. Findings from this study may help improve patient comfort during SPLM and could inform the teaching of SPLM, helping both the student learner and student patients.

### Primary hypothesis

In adults with symptomatic LBP, the application of two distinct SPLM procedures will result in significantly different levels of reported comfort.

### Secondary hypothesis

Directionality of LBP (e.g., flexion vs. extension) is associated with the comfort reported during two types of SPLM procedures.

## Methods

This was a crossover randomized controlled study. CONSORT checklist for randomized crossover RCT’s was used to assist in the design of this study and manuscript preparation. A crossover design was chosen since changes in comfort during SPLM are typically short-lived and the design removes between-group variation. In addition, some of the known disadvantages of the crossover design (e.g. larger dropout rate, changes in the patient’s condition, and a potential carryover effect) were not expected in this study. A 10-min washout period was chosen to allow enough time for any transient changes in comfort to have faded before the next procedure. The protocol for this study was retrospectively registered on Open Science Framework (10.17605/OSF.IO/796EB). No changes were made to the collection protocol after trial commencement. Post collection deviations included an exploratory analysis of within-participant differences.

Participants were recruited by video advertisement on social media (Facebook) and direct recruitment (word of mouth) through clinicians and interns at the Canadian Memorial Chiropractic College (CMCC) campus clinic (Toronto, Canada) between October and December 2021. This study was approved by the CMCC’s Research Ethics Board and all participants provided written informed consent prior to participating in the study.

### Participants

To participate in the study, inclusion criteria were: adults aged between 18 and 60 years old, being a student or faculty member at CMCC, experiencing LBP on the day of collection (≥ 2/10 on an 11-point Numeric Rating Scale [NRS]), where 0 corresponded to the most comfortable and 10 corresponded to the most uncomfortable). Exclusion criteria were: treatment to the lumbo-pelvic region in the previous 24 h (e.g. SMT/mobilization, massage, yoga, physical modalities, etc.), surgery or injection to the lumbopelvic region in the last 2 months, recent or ongoing hip pathology (e.g. femoroacetabular impingement, moderate-severe osteoarthritis, etc.), lumbar, pelvic or hip fracture or trauma in the last 2 months, infection or tumor of lumbopelvic region in the last 5 years, pregnancy, currently on medications that alter pain (non-steroidal anti-inflammatory drugs, opioids, antidepressants, etc.), leg dominant pain that is worse than the back pain, bilateral arm or leg pain and weakness, saddle anaesthesia, changes in bowel or bladder function in the previous 3 weeks, recent night pain, fever, chills or night sweats in the last 3 weeks, or recent unexplained weight loss.

### Randomization

Order of SPLM procedure was randomized. The randomization scheme was prepared by the study biostatistician (SHJ) employing mixed blocks of size 2, 4 and 6. The order of manipulation procedure for each subject was sealed in sequentially numbered opaque envelopes, which were prepared prior to data collection by the same author (SHJ), who was not involved in data collection. For each newly enrolled eligible, consenting participant, the next envelope in sequence was opened by experimenter 2. Experimenter 1 was blinded to the order of SPLM position.

### Study protocol

Data collection took place in the Human Performance Lab at CMCC. Experimenter 1 conducted the informed consent, eligibility screening (inclusion/exclusion criteria), administered the intake questions (age, sex, height, weight, duration of LBP), and recorded the baseline comfort scores on an 11-point NRS. Participants were asked to “Please rate your low back comfort on a scale from 0 to 10, 0 being the most comfortable, 10 being the most uncomfortable”. Scores were recorded during quiet standing, standing maximal active lumbar flexion, extension, and bilateral rotation. The rotation direction with less range was subjectively noted, when present. Experimenter 1 then left the lab and informed experimenter 2 (licensed chiropractor with 15 years’ experience) of the side with the least rotation, which was recorded by experimenter 2 and determined the upside for SPLM procedures. The least rotation side was chosen to limit both procedures to the same side, which avoided confounding the data by side. If both rotation directions were equal, the upside was determined by the most tender location reported by the participant during examination (described in detail below), which was also used to determine the target spinal level. Experimenter 2 entered the lab, examined and recorded the most tender location during palpation of the lumbar mamillary processes and the posterior superior iliac spine (PSIS) in a posterior to anterior direction, in the prone position. Experimenter 2 was blinded to the comfort scores.

**Fig. 1:**
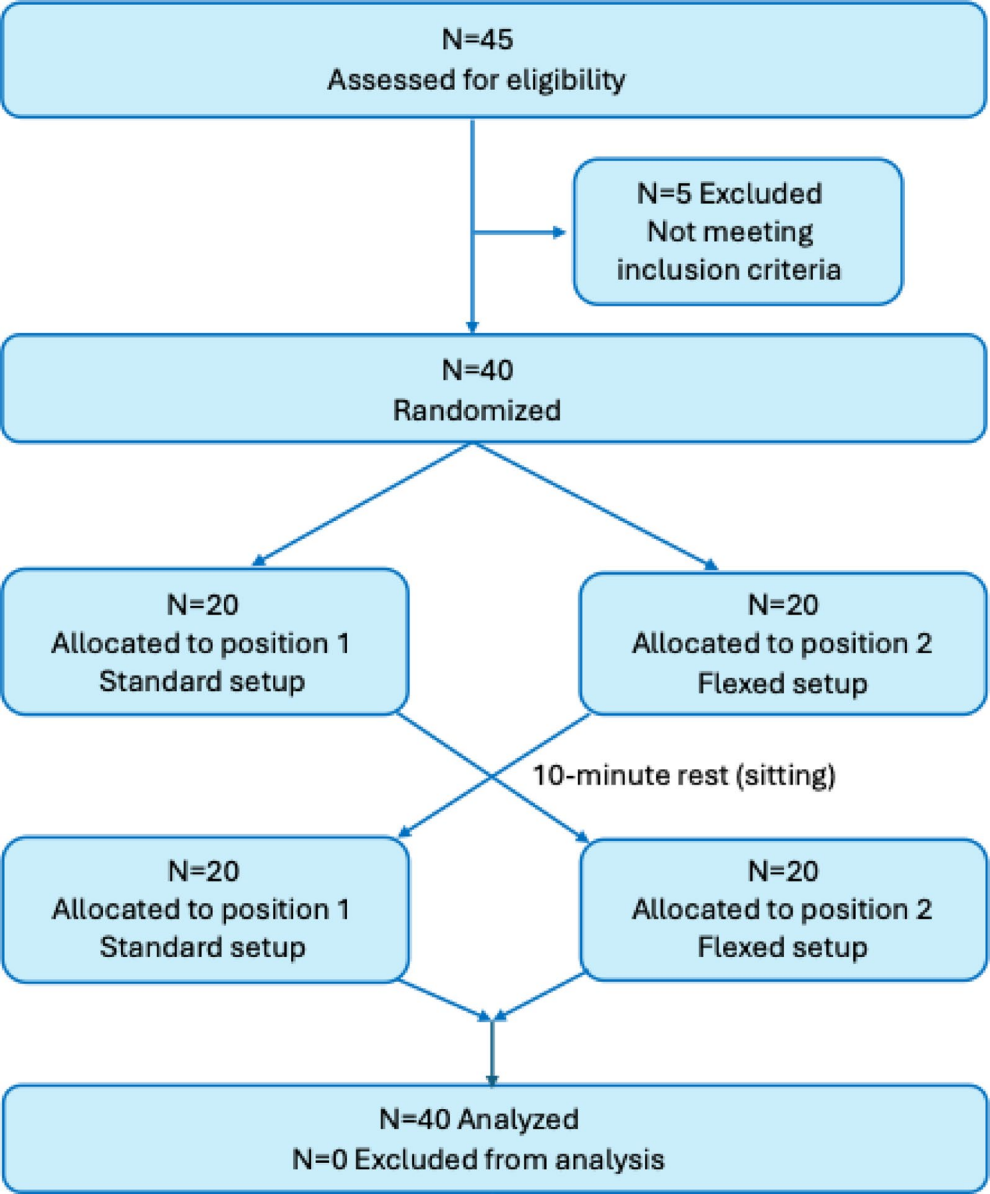
Participant flow chart

Prior to delivering the first high velocity low amplitude SPLM, experimenter 2 opened the next opaque envelope in sequence to reveal the order of procedures to be used. Each participant received the Standard high velocity low amplitude SPLM (hypothenar/ilium or mamillary push) and the Modified flexed SPLM in random order, on the same side, with a 10-min sitting rest period in between (see Fig. 1). After each manipulation, experimenter 2 recorded if an audible cavitation was heard and left the room. Experimenter 1 re-entered to collect comfort scores and any locations of discomfort. Participants were asked to “Please rate your comfort during manipulation on a scale of 0–10, 0 being the most comfortable and 10 being the most uncomfortable.” If a score greater than 0 was reported, Experimenter 1 asked the follow up question: “Where did you feel the discomfort?” and recorded the responses.

### Interventions

**Fig. 2:**
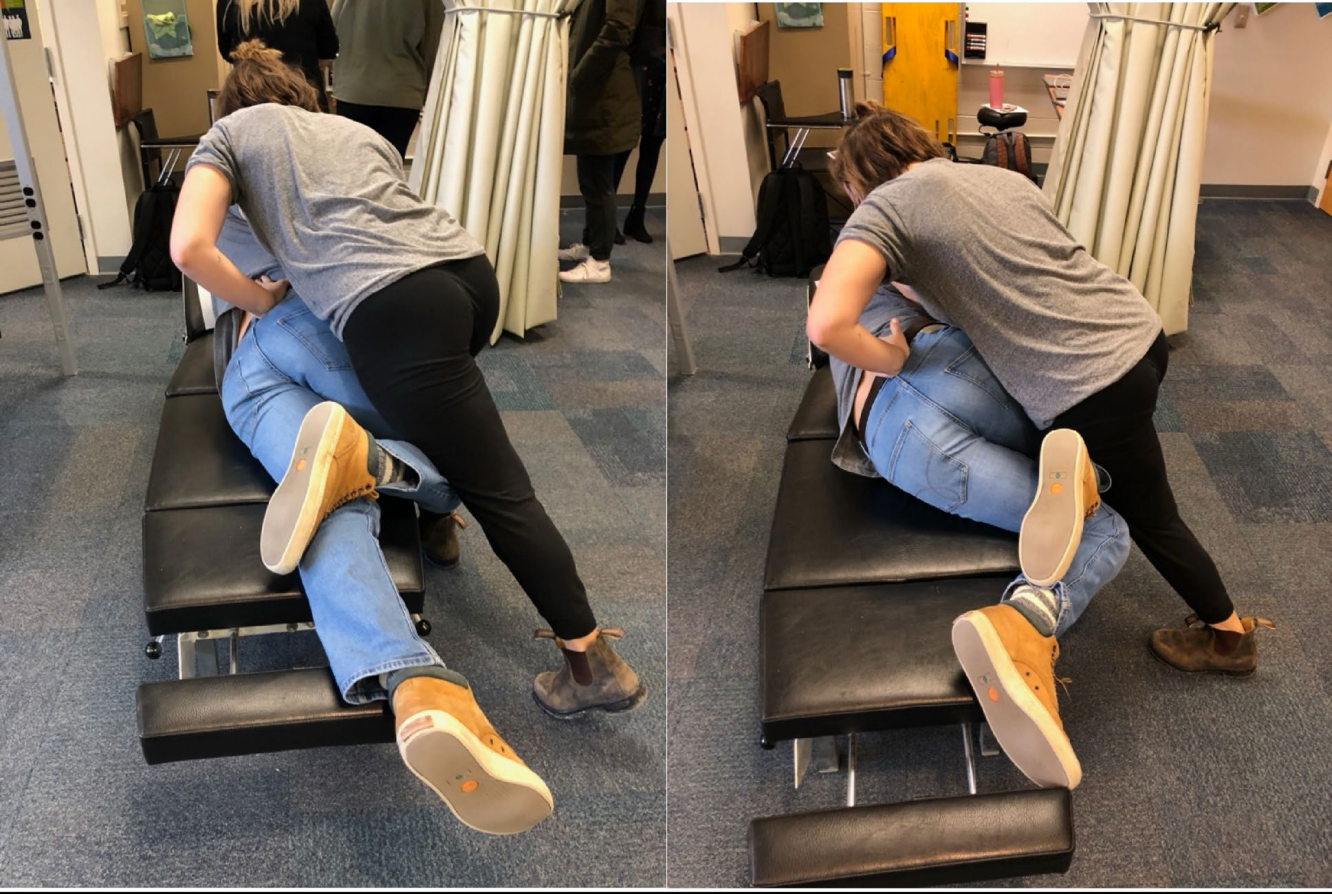
Standard SLPM setup (left). Modified flexed SPLM setup (right)

The setup procedure for the Standard SPLM started by asking the participant to lay down on their side, facing the experimenter and cross their arms in front of their chest. The experimenter brought the upside leg to approximately 90° hip flexion and slightly rotated torso/shoulders backwards, with the pelvis rotated forwards, until the frontal plane of the pelvis was approximately 45° off vertical. The experimenter contacted the participant’s most tender lumbar or sacroiliac segment with a hypothenar contact, while stabilizing the torso with the non-contact hand on the participants arms. Pressure was applied on the spinal segment until end-range preload rotation was reached (Fig. 2). A high velocity, low amplitude thrust was then applied. The Modified flexed position was similar to the Standard setup, but with an increase in hip and lumbopelvic flexion. The participant’s upside leg was moved towards end-range hip flexion, until resistance to more flexion was felt, and increased lumbopelvic flexion was observed. The downside hip was also flexed to facilitate lumbopelvic flexion (Fig. 2).

### Sample size calculation

For paired t-test comparing Standard SPLM to Modified Flexed SPLM, with significance α = 0.05, power 1-β = 0.80, a sample size of 40 participants was determined to be sufficient to detect an effect size of 0.4 which is in the small to moderate range according to Cohen’s *d* [12].

### Data analysis

Descriptive data on demographics and baseline comfort scores were compiled, mean and standard deviations (SD) were calculated. Descriptive data on spinal target level and clinician reported audible cavitations were compiled by Experimenter 1. Descriptive data on reported locations of discomfort were compiled and coded by Experimenter 2. The mean comfort scores between each procedure and between the order of procedures were calculated and compared via paired t-tests, following the protocol for crossover studies described by Fleiss [17]. In addition, Cohen’s *d* effect size (*d* = mean difference/SD) and 95% Confidence Intervals (95%CI) were computed for the differences in comfort between procedures and between order. A secondary analysis was also completed to examine correlations between comfort during standing flexion, extension, rotation, and the two SPLM procedures. An exploratory observational analysis to compare within-participant differences was completed. Participants whose comfort score differed by 2.0 points or more between the two procedures were identified. For these participants, we counted the number who reported lower scores for the Standard SPLM and the number who reported lower scores for the Modified Flexed SPLM. A minimum clinically important difference (MCID) of 2.0, previously used for pain, was used to interpret differences in comfort scores [9, 30]. Within-participant values were plotted for the two procedures as a spaghetti plot.

## Results

Forty-five participants were recruited of which 5 were excluded due to medications that can affect pain (Muscle relaxants and Selective Serotonin Reuptake Inhibitors) (Fig. 1).

Twenty participants were allocated to each intervention sequence and there were no dropouts. Nineteen participants received SPLM with their left side up and 21 with their right side up. Data collection ended when the pre-determined sample size was reached (n = 40). Participant characteristics are presented in Tables 1 and 2.

**Table 1:** Participant characteristics

	Sequence A (n = 20) (Standard to modified flexed)	Sequence B (n = 20) (Modified flexed to standard)	Total (n = 40)
*Characteristic mean (SD), min–max*			
Age (years)	26 (3.5), 22–36	25.1 (1.8), 22–28	25.5 (2.9), 22–36
Height (inch)	66.9 (3.8), 61–70	66.2 (3.2), 60–70	66.9 (3.8), 60–70
Weight (lbs)	156.8 (27.1), 122–220	149.2 (22.4), 118–200	153 (24.9), 118–220
Baseline comfort (NRS)	3.2 (1.8), 1–9	2.8 (1.1), 1–5	3.0 (1.5), 1–9
*Position—comfort (NRS) mean (SD), min–max*			
Standing	2.1 (1.8), 0–7.5	1.6 (1.3), 0–5	1.9 (1.6), 0–7.5
Flexion	2.5 (1.5), 0–6	1.7 (1.1), 0–3.4	2.1 (1.4), 0–6
Extension	3.1 (1.9), 0–6	3.3 (1.7), 0–7	3.2 (1.8), 0–7
Right rotation	2.1 (1.9), 0–8	2.1 (1.5), 0–5	2.1 (1.7), 0–8
Left rotation	2.2 (2.2), 0–8	1.9 (1.5), 0–5	2.1 (1.9), 0–8

*SD* standard deviation, lbs (pounds), *NRS* numeric rating scale

**Table 2:** Participant categorical characteristics

Category	Sequence A (n = 20) (Standard to modified flexed)	Sequence B (n = 20) (Modified flexed to standard)	Total (n = 40)
*Sex, n (%)*			
Female:	14 (70%)	16 (80%)	30 (75.0%)
Male:	6 (30%)	4 (20%)	10 (25.0%)
*LBP chronicity (weeks) n (%)*			
< 3	4 (20%)	4 (20%)	8 (20.0%)
3–12	1 (5%)	4 (20%)	5 (12.5%)
> 12	15 (75%)	12 (60%)	27 (67.5%)

*LBP* low back pain

**Fig. 3:**
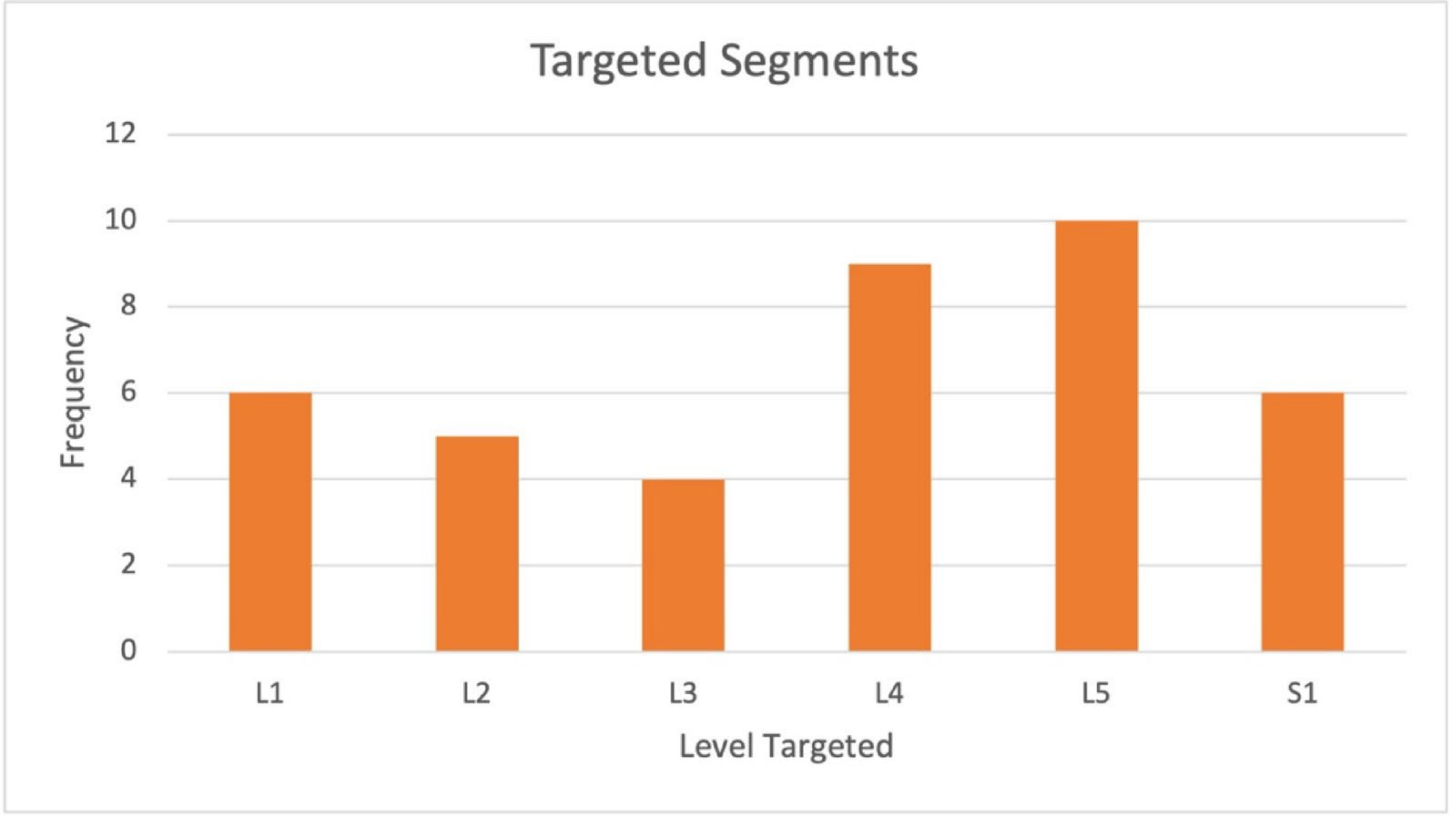
Frequency of targeted thrust location during SPLM

Baseline characteristics between groups (initial procedures) are similar, reducing the likelihood of results being influence by baseline imbalance. Targeted segments ranged from L1 to S1, with the most frequent target being at the level of L5 (see Fig. 3).

Audible cavitations were more frequently recorded during the first procedure (80%), compared to the second procedure (40%) regardless of the order of SPLM, and were equally common on the left and right sides.

**Fig. 4:**
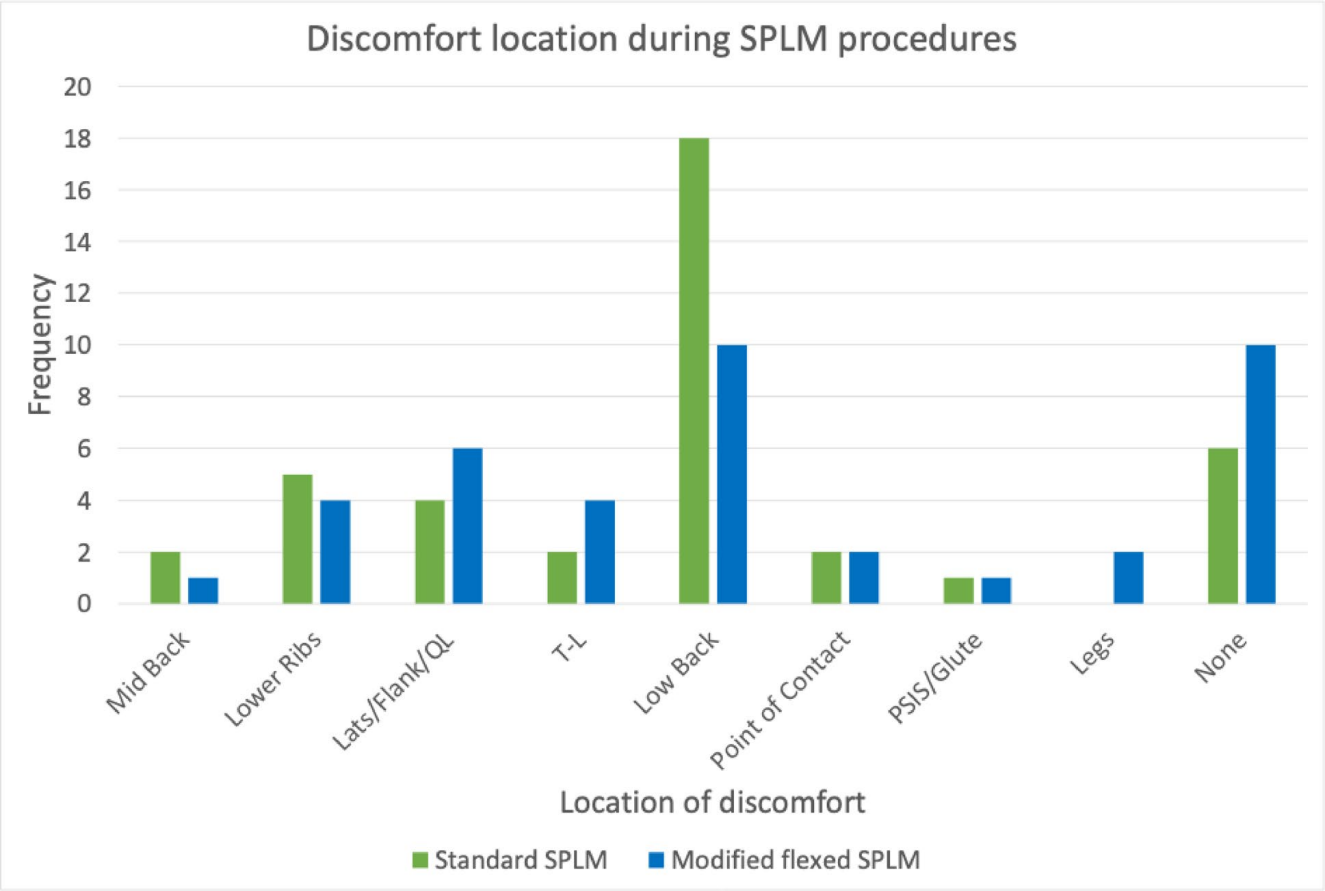
Location of reported discomfort. SPLM: side posture lumbopelvic manipulation

The reported location of discomfort varied, with the highest frequency of complaints located in the low back (see Fig. 4). Thirty-four harms were reported, all of which were mild in nature (e.g. uncomfortable). Reports greater than “0” were 31/40 for Position 1 and 34/40 for position 2, on the comfort scale.

No differences in comfort were found between the Standard SPLM versus the Modified flexed SPLM and the effect size was very small (Table 3). A common limitation to cross-over study designs is the potential carryover effects of the previous intervention. Since the washout period was relatively short (10 min), an analysis of comfort scores between the first and second procedure was completed, showing no significant differences between groups (Table 3). This suggests a carryover effect for comfort was unlikely.

**Table 3:** Comfort scores

Description	Mean comfort (SD)	Min–Max	t-stat, df, p-value	Effect size Cohen’s *d* (95% CI)
SPLM—Standard	2.2 (1.9)	0–8		
SPLM—Modified flexed	2.3 (2.4)	0–9		
Difference between standard & flexed	− 0.01 (2.3)	− 8–5	− 0.03, 39, 0.97	*d* = − 0.004 (− 0.32, 0.32)
First SPLM	2.3 (2.3)	0–9		
Second SPLM	2.2 (2.0)	0–8		
Difference between 1st and 2nd SPLM	0.04 (2.3)	− 4–8	0.10, 39, 0.92	*d* = 0.016 (− 0.30, 0.34)

*SPLM* side posture lumbopelvic manipulation, *SD* standard deviation, *t-stat* paired t statistic, *df* degrees of freedom

No correlations were found between the comfort scores during AROM testing and the two SPLM procedures (Table 4).

**Table 4:** Correlation analysis between SPLM procedure comfort scores and baseline comfort scores

Pearson correlation coefficient (r) Prob >|r| under H0: Rho = 0 (p) (n = 40)		
Baseline comfort score	Standard SPLM	Modified flexed SPLM
Standing	r = 0.002 *p* = 0.99	r = − 0.039 *p* = 0.81
Max flexion AROM	r = 0.036 *p* = 0.82	r = − 0.107 *p* = 0.51
Max extension AROM	r = 0.057 *p* = 0.73	r = − 0.066 *p* = 0.68
Max right rotation AROM	r = 0.065 *p* = 0.69	r = 0.068 *p* = 0.68
Max left rotation AROM	r = − 0.039 *p* = 0.81	r = − 0.096 *p* = 0.56

*SPLM* side posture lumbopelvic manipulation, *AROM* active range of motion

**Fig. 5:**
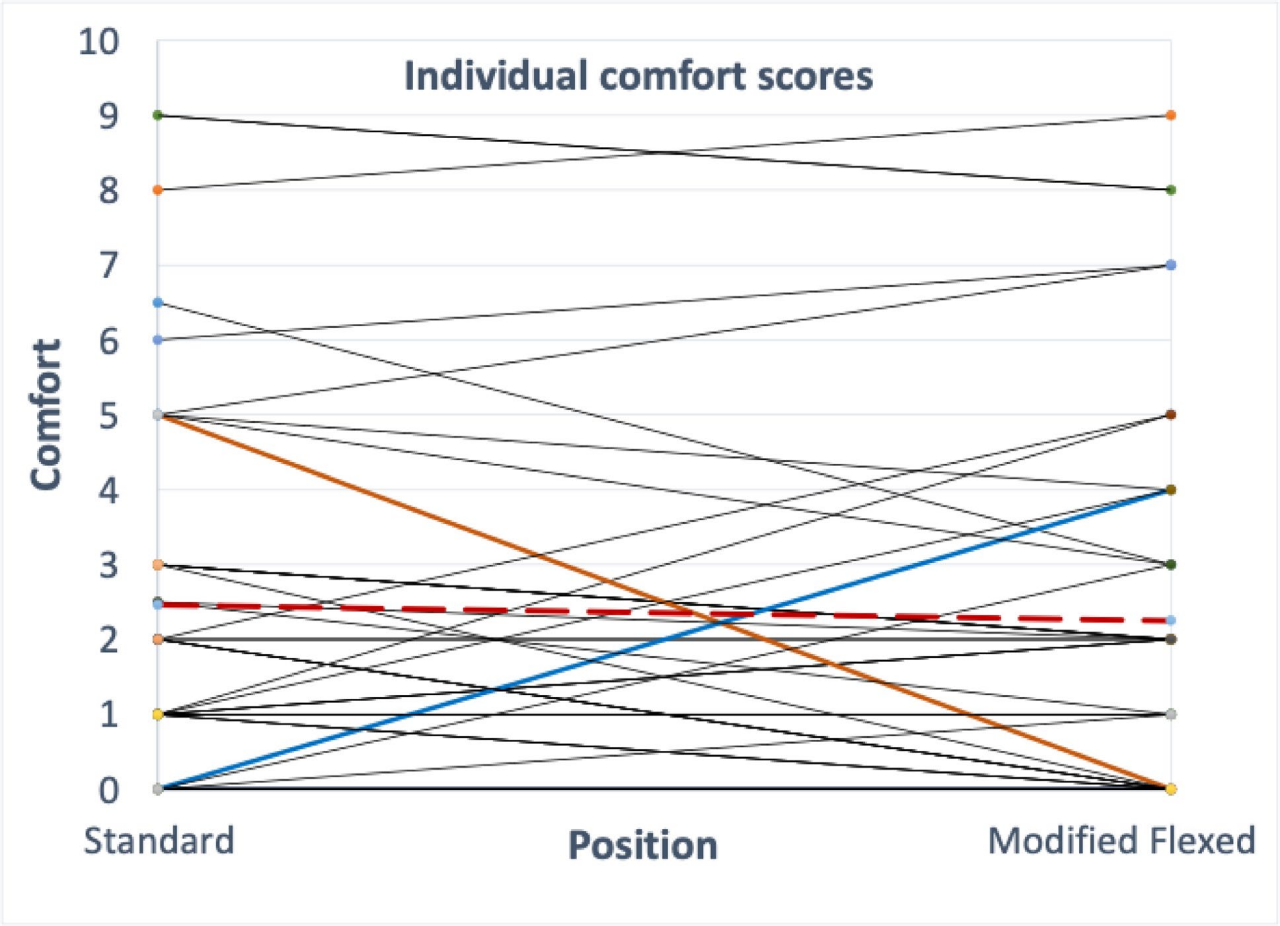
Within-participant comparisons for each participant’s comfort scoring between the standard and modified flexed SPLM. Dashed line represents the mean within-participant comparison across all participants. Two thick lines represent individual participants who had an obvious difference in comfort between the two procedures. Standard: Standard side posture lumbopelvic manipulation, Modified: Modified Flexed side posture lumbopelvic manipulation

From an exploratory analysis observing within-participant comfort scores between each SPLM procedure, six participants rated at least 2/10 lower comfort scores for the standard SPLM and eight rated at least 2/10 lower comfort scores for the modified flexed SPLM (see Fig. 5).

## Discussion

Tailoring treatment to meet the needs of patients is a fundamental concept in health care. This study compared two versions of a side posture lumbopelvic manipulation and found no difference, on average, in comfort ratings between a Standard and a Modified flexed SPLM. One potential factor that could influence this finding is the lack of population subgrouping. Subgrouping LBP patients based on their presentation of direction related LBP has been recommended to improve patient outcomes and is being used in LBP care pathways [2, 20, 24, 31]. This study also examined the relationship between comfort during standing maximal active lumbar flexion, extension, rotation, and the two SPLM procedures, however no relationships were found. This finding could be due to our sample inclusion criteria. Previously reported relationships between extension related LBP and matching treatments with flexion or extension had more stringent inclusion criteria. For example, Curran and colleagues looked at matching recommendations for seat pan inclination for a population with extension-related low back pain and found that low back discomfort was worse on average when sitting for 10 min with a forward-inclined seat pan [14]. Seeing no difference in this study’s results could be due to the non-specific low back pain population studied as well as the duration of the intervention (much shorter than 10 min of sitting).

Examining the within-participant comparisons suggests that some people do have a comfort preference for how much lumbopelvic flexion is applied during SPLM. Since patient comfort has been suggested to be a predictive factor in success of treatment with SMT [27], determining variations and modifications for treatment application, that can alter comfort, may be beneficial for some.

In determining how to improve comfort during SMT, it may also be important to understand the location of discomfort. Our results suggest that the location of discomfort can vary between participants, with the low back region being the most commonly reported. This finding is not surprising considering the heterogeneity of the LBP population, however approximately half of the participants reported discomfort away from the low back, which suggests that different strategies to improve comfort may be needed depending on where the discomfort is.

Limitations of this study include the broad inclusion of LBP types and the lack of subgrouping participants based on reported aggravation of symptoms. This study did include an initial analysis of direction related comfort; however, this single assessment may not have been sufficient to observe an extension or flexion related LBP pattern, reported by other authors [15].

The sample of participants was limited to students and faculty of a chiropractic college, due to pandemic restrictions, which limits the generalizability of these findings.

Participants were not blinded to the procedure they received due to the physical nature of the procedures, which creates a potential bias in participant reporting.

The numeric rating scale used in this study has not been validated. 11-point numeric pain rating scales are common and have been validated [1, 3, 38], however there were no validated numeric comfort rating scales at the time of this study. Similarly, the 2/10 MCID chosen for this study was also not validated. This value was chosen from previously published MCID scores on pain. It is also important to note that there are many aspects of the patient experience that were not measured in this study (e.g. anxiety, fear, overall acceptability), which can alter an individual’s perception of the procedure.

Another limitation is the lack of standardized position for the Modified flexed SPLM procedure. No kinematic measurements of lumbopelvic flexion were used to standardize the modified flexed position. However, by flexing each participant towards their end range where resistance was felt by the experimenter, the Modified flexed setup position was relative to each participants passive flexibility. It is also possible that a moderate or in-between amount of flexion would have been preferred by some of the participants.

## Conclusion

Comparing comfort during a modified flexed side posture lumbopelvic manipulation to a standard one revealed no difference for a population with low back pain. However, some individuals may have differences in comfort during a Standard or a Modified flexed setup position. Future studies should examine other variations of manipulation for particular subgroups of people with low back pain.

## Data Availability

The datasets used and/or analysed during the current study are available from the corresponding author on reasonable request.

## Abbreviations

LBP: Low back pain
SMT: Spinal manipulation therapy
CPR: Clinical prediction rules
SPLM: Side posture lumbopelvic manipulation
NRS: Numeric rating scale
PSIS: Posterior superior iliac spine
L1: First lumbar vertebra
L5: Fifth lumbar vertebra
S1: First sacral vertebra
SD: Standard deviation
t-stat: Paired t statistic
df: Degrees of freedom

## Declarations

### Trial registration on open science framework

Our study was retrospectively registered. We began planning for this study in September 2020. Our planning was derailed due to ongoing changes to collecting data with live human participants due to COVID pandemic safety protocols, where we were unsure if our study would be allowed to proceed. When we finally received approval to collect data, we focused on ensuring the study protocol was following COVID protocols, and the study protocol registration was unintentionally oversighted. We recognize the potential for lack of transparency and the challenge a journal would have with distinguishing between confirmed and unexpected findings. However, we have clearly stated where exploratory analysis was completed and have uploaded our original research ethics board proposal to the Open Science Framework (10.17605/OSF.IO/796EB), which outlines our full trial protocol, methods and data analysis. We are happy to provide the journal editors with the original proposal documents that have the time stamps from 2020 and 2021.
